# Aberrant Coupling Between Resting-State Cerebral Blood Flow and Functional Connectivity in Wilson’s Disease

**DOI:** 10.3389/fncir.2019.00025

**Published:** 2019-04-18

**Authors:** Sheng Hu, Hongli Wu, ChunSheng Xu, Anqin Wang, Yi Wang, Tongping Shen, Fangliang Huang, Hongxing Kan, Chuanfu Li

**Affiliations:** ^1^Medical Information Engineering, Anhui University of Chinese Medicine, Hefei, China; ^2^Laboratory of Digital Medical Imaging, Medical Imaging Center, The First Affiliated Hospital of Anhui University of Chinese Medicine, Hefei, China

**Keywords:** neurovascular coupling, basal ganglia, arterial spin labeling, cerebral blood flow, functional magnetic resonance imaging

## Abstract

Both abnormalities of resting-state cerebral blood flow (CBF) and functional connectivity in Wilson’s disease (WD) have been identified by several studies. Whether the coupling of CBF and functional connectivity is imbalanced in WD remains largely unknown. To assess this possibility, 27 patients with WD and 27 sex- and age-matched healthy controls were recruited to acquire functional MRI and arterial spin labeling imaging data. Functional connectivity strength (FCS) and CBF were calculated based on standard gray mask. Compared to healthy controls, the CBF–FCS correlations of patients with WD were significantly decreased in the basal ganglia and the cerebellum and slightly increased in the prefrontal cortex and thalamus. In contrast, decreased CBF of patients with WD occurred predominately in subcortical and cognitive- and emotion-related brain regions, including the basal ganglia, thalamus, insular, and inferior prefrontal cortex, whereas increased CBF occurred primarily in the temporal cortex. The FCS decrease in WD patients was predominately in the basal ganglia and thalamus, and the increase was primarily in the prefrontal cortex. These findings suggest that aberrant neurovascular coupling in the brain may be a possible neuropathological mechanism underlying WD.

## Introduction

Wilson’s disease (WD) is an inherited disorder of copper metabolism. The symptoms were first identified by Dr. Samuel Alexander Kinnier Wilson in 1912 (Wilson, 1912). The physical burden of the disease falls on the liver and the brain. Copper deposition in the brain affected by WD occurs primarily in the basal ganglia. In early stages of the disease, this results in neurological or psychiatric symptoms, such as mild cognitive deterioration, whereas late stages are characterized by dysarthria (de Bie et al., 2007), abnormal gait, risus sardonicus, dystonia, rigidity, and dyskinesia (Machado et al., 2006; Litwin et al., 2016). The neuropathological characteristics of WD are neuronal loss and atrophy in the bilateral basal ganglia, brainstem, cerebellum, and cerebral cortex (Page et al., 2004).

Resting-state functional connectivity (rsFC), which measures the temporal correlations of low-frequency fluctuations in the blood-oxygen-level-dependent (BOLD) signal across the brain regions (Biswal et al., 1995), has provided evidence that copper deposition profoundly impairs intrinsic brain activity (Hu et al., 2017). Seed-based FC analysis has been used to quantify FC changes in WD and has uncovered abnormal FC in the default mode network (DMN) (Han et al., 2016). Although these methods are conducive to quantifying functional dysconnectivity in the brain of individuals with WD, both rsFC and seed-based FC analysis have issues. Seed-based FC methods require *a priori* knowledge of the seed region. Thus, it is difficult to analyze data if the underlying pathology of a disease is unknown (Nair et al., 2014). Low-frequency BOLD signals are susceptible to physiological processes, such as respiratory and cardiac oscillations, which may lead to unreliable results when using rsFC methods (Biswal, 2012). Functional connectivity strength (FCS) analysis is a new data-driven method to measure average FC by calculating the correlation of each voxel with all other voxels in the brain (Liang et al., 2013). Brain regions with high FCS are identified as functional hubs, which have high connectivity to the remainder of the brain.

In the resting brain, cerebral blood flow (CBF) is closely related to glucose utilization, oxygen consumption, and aerobic glycolysis (Vaishnavi et al., 2010). Some studies have noted CBF deficits in WD. For example, positron emission tomography (PET) and single-photon emission computed tomography (SPECT) have revealed decreased metabolic rate of glucose consumption and hypoperfusion in the prefrontal cortex, occipital cortex, cerebellum, striatum, and thalamus (Hawkins et al., 1987; Kuwert et al., 1992). However, these two techniques are invasive, with a long acquisition time and low spatial resolution. Arterial spin labeling (ASL) MRI is a non-invasive technique for measuring CBF using an endogenous contrast (Roberts, 1994; Golay et al., 2004). Therefore, it has been widely used to evaluate cerebrovascular disease, including arterio-occlusive disease, and as a biomarker for neuronal metabolism in other disorders, such as Alzheimer’s and Parkinsonism diseases (Telischak et al., 2015).

The human brain accounts for approximately 20% of the body’s total energy demands (Raichle and Gusnard, 2002), which is more than any other organ. Brain metabolism, which consumes most of the glucose and produces most of the energy, is critical for supporting spontaneous brain activity (Raichle and Mintun, 2006). Brain regions with stronger neuronal activity tend to have greater brain metabolism, resulting in increased CBF (Kuschinsky, 1991; Venkat et al., 2016). The strength of neuronal activity in one brain region reflects the degree of functional connectivity with other brain regions (Zhu et al., 2017). Therefore, the degree of functional connectivity is related to brain metabolism, especially glucose utilization (Tomasi et al., 2013). Several studies have identified the relationship between functional networks and CBF. For instance, FCS analysis in healthy subjects and those with neurological diseases has suggested that brain FCS is coupled with CBF (Liang et al., 2013; Zhu et al., 2017).

Patients with WD experience copper accumulation in the brain, neuronal loss, and atrophy (Ala et al., 2007; Huster, 2010), possibly resulting in neurovascular imbalance. Therefore, we hypothesized that the coupling between CBF and brain FC would be abnormal in patients with WD relative to healthy subjects. To verify this hypothesis, we collected resting-state functional MRI (fMRI) data and ASL data from 27 patients with WD and 27 sex- and age-matched healthy controls to measure the brain FCS and CBF. Further, FCS–CBF correlations were compared between the groups.

## Materials and Methods

### Subjects

Twenty-seven patients with WD with neurological symptoms (mean age, 22.42 years; SD, 3.66 years; 12 females) were recruited from the First Affiliated Hospital of Anhui University of Chinese Medicine (AUCM), and 27 age- and sex-matched healthy controls (mean age, 23.51 years; SD, 2.67 years; 12 females) were recruited from the local community. All patients were receiving drug treatment, including penicillamine and zinc salts. WD diagnosis was based on clinical symptoms (e.g., presentation of extrapyramidal and pyramidal symptoms and signs, and behavioral problems), the presence of the Kayser–Fleischer (KF) ring, and abnormal copper metabolism with serum ceruloplasmin (CP) less than 20 mg/dl and 24-h urinary excretion of copper of more than 1.6 μmol/day. The average CP was 6.28 ± 2.40 mg/dL (mean ± SD), and the average 24-h urinary copper excretion was 2.52 ± 1.44 μmol/day based on the WD group. There were no neurological diseases other than WD in the patients. Healthy controls had no history of head injury, neurological disorder, or concomitant medical disorder. All participants provided written informed consent. This study was approved by the Human Research Committee of the First Affiliated Hospital of AUCM. Detailed information regarding the subjects is presented in Table 1.

**Table 1:** Characteristics of the participants.

	WD (*N* = 27)	HC (*N* = 27)
Gender (male/female)	15/12	15/12
Age (years)	18–28 (22.42 ± 3.66)	19–31 (23.51 ± 2.67)
Education (years)	8–14 (10.21 ± 1.23)	9–15 (12.32 ± 2.55)
Handedness	27 right-handed	27 right-handed
WD duration (years)	4–10 (6.76 ± 1.38)	
WD types	Neurologic	
The KF ring	27 WD with the KF ring	
24-h urinary Cu (μmol/day)	1–6 (2.52 ± 1.44)	
CP (mg/dl)	3.52–13.24 (6.28 ± 2.40)	


*WD, Wilson’s disease; HC, healthy controls; N, number, KF, Kayser–Fleischer, Cu, copper, CP, ceruloplasmin*.

### Data Acquisition

MRI data were acquired using a 3.0-Tesla MR system (Discovery MR750, General Electric) with an eight-channel high-resolution radio-frequency head coil. Sagittal 3D T1-weighted images were acquired using a T1-3D BRAVO sequence with repetition time (TR)/echo time (TE) of 8.16 ms/3.18 ms, a flip angle (FA) of 12°, a 256 mm × 256 mm matrix, a field of view (FOV) of 256 mm × 256 mm, and a slice thickness of 1 mm, with 200 axial slices with no gap. Resting-state fMRI images were acquired using a gradient-echo single-shot echo planar imaging sequence with a TR/TE of 2,000/30 ms, an FOV of 220 mm × 220 mm, a 64 × 64 matrix, an FA of 90°, and a slice thickness of 3 mm, with 185 volumes. Functional ASL data were acquired using a pseudocontinuous arterial-labeling (pCASL) technique. Interleaved control and label images were acquired using a gradient echo EPI sequence with a TR/TE of 5,046/11 ms, an FA of 111°, 50 slices with 3-mm thickness, an FOV of 220 mm × 220 mm, a 128 × 128 matrix, three excitations, a post-label delay of 2,025 ms, and a spiral-in readout of 8 arms with 512 sample points. All participants were asked to keep their eyes closed, relax, move as little as possible, think of nothing, and stay awake during the scans.

### Functional Magnetic Resonance Imaging Data Preprocessing

All fMRI data preprocessing was performed using SPM12^1^ based on batch scripts. To account for magnetization equilibrium and subject’s adaptation to the experimental environment, the first 10 volumes were removed. The remaining images underwent slice timing correction to address the time delay between different slices, and then all images were realigned to the first volume to correct for head motion. Subjects were excluded if head motion exceeded 1 mm of displacement or 1° of angular rotation. After head motion correction, the functional images were co-registered to the T1-weighted anatomical images, followed by removing white matter, and cerebrospinal fluid signals. Then, the functional images were normalized to the MNI152 standard brain. To reduce low-frequency drift (0.008 < *f* < 0.1) and Gaussian noise, all fMRI data were band-pass-filtered and spatially smoothed using a 6-mm full-width at half-maximum Gaussian kernel (FWHM).

### Whole-Brain Functional Connectivity Strength Analysis

The FCS analysis was conducted using the Degree Centrality package of DPARSF^2^. For resting-state data, the BOLD time course of each voxel within gray matter (GM) was first extracted, and then correlation coefficients between all pairs of voxels within the GM were calculated. In order to eliminate weak correlations possibly arising from background noise, we restricted our analysis to positive correlations above a threshold of 0.25 (Liang et al., 2013; Wang et al., 2014; Liu et al., 2015). The correlation threshold was applied to FCS analysis to decrease the impact of false-positive connectivity on the data. The voxel was set to zero if its functional connectivity was smaller than the threshold. FCS at a given voxel x0 was calculated as the average of functional connectivity between x0 and all other voxels in the brain. Voxels with high FCS exhibited high connectivity to the remainder of the brain. The FCS maps were finally spatially smoothed using a 6-mm FWHM Gaussian kernel.

### CBF Data Analysis

The ASL data were analyzed offline using the ASL Data Processing Toolbox (Wang et al., 2008). Perfusion-weighted image series were generated by subtraction of the label and control images, followed by conversion to absolute CBF images based on a single-compartment continuous ASL perfusion model (Wang et al., 2005). The CBF maps were first removed of non-brain tissue and co-registered to T1-weighted anatomical images. Then, the CBF image of each subject was normalized to the MNI152 standard brain and spatially smoothed using a 6-mm FWHM.

### Cerebral Blood Flow – Functional Connectivity Strength Coupling Analysis

For each participant, both CBF and FCS values were standardized to *z* scores so that they could be averaged and compared across subjects. To quantitatively measure the coupling between CBF and FCS, we performed correlation analyses across voxels and subjects. Because neighboring voxels could be highly dependent, due to physiological correlations and spatial preprocessing, such as registration and spatial smoothing, the effective degrees of freedom (dfeff) in across-voxel correlation analysis was much smaller than the number of voxels used in the analysis. The dfeff of across-voxel correlations was estimated using the same method as proposed by Liang et al. (2013) in their CBF–FCS coupling study. The dfeff of across-voxel correlations was 1,342, and the number of voxels was 271,633 in this study. The CBF–FCS correlation coefficient value reflected the consistency of the spatial distribution between CBF and FCS.

Basal ganglia are directly or indirectly connected to the cerebellum, thalamus, and prefrontal cortex, and these connections may be mediated by the cortico-basal ganglia-thalamo-cortical circuit (CBGTC). Therefore, CBF–FCS coupling was also calculated in these regions, as extracted from the AAL template that was used widely by brain image researchers (Dimitriadis et al., 2017; Zou et al., 2018). Given the difference between the AAL template and the standard MNI template used in this study, we firstly co-registered the AAL template to the standard GM mask and further mask it with the standard GM mask. Finally, we extracted the mask for CBF–FCS coupling analysis.

### Group Analysis

Across-subjects average CBF and FCS *z*-transformed maps were computed to demonstrate the spatial distribution patterns. A group-level two-sample *t* test was applied to identify the intergroup differences in CBF and FCS, and the whole brain correction was applied *via* Monte Carlo simulations (3dClusSim, AFNI package) at a voxel-wise height threshold of *p* < 0.001 and a cluster size threshold of 30 contiguous voxels.

## Results

### Spatial Distribution of Functional Connectivity Strength and Cerebral Blood Flow

**FIGURE 1:**
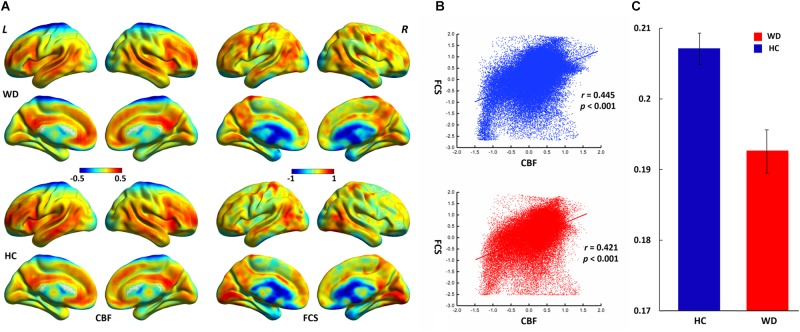
Spatial distribution and neurovascular coupling in CBF and FCS. **(A)** Spatial distribution maps of CBF and FCS. **(B)** The whole-brain CBF–FCS coupling changes across voxel in the WD. **(C)** The whole-brain CBF–FCS coupling changes across subject in the WD. The FCS and CBF maps are normalized to *z* scores and averaged across subjects within groups. L, left; R, right. The right picture is whole gray matter (GM) level CBF–FCS coupling changes in WD. Scatter plots of the mean GM level spatial correlations across voxels between CBF and FCS in HC (blue) and WD (red), respectively. The mean CBF–FCS correlation across subjects in WD and HC. CBF, cerebral blood flow; FCS, functional connectivity strength; HC, healthy controls; WD, Wilson’s disease.

The WD group and healthy controls showed similar spatial distributions of FCS and CBF (Figure 1). Higher CBF was primarily distributed among the posterior cingulate cortex, precuneus, prefrontal cortex, lateral temporal cortex, and DMN. Higher FCS was primarily observed in the visual cortex, prefrontal cortex, posterior cingulate cortex, and DMN.

### Functional Connectivity Strength and Cerebral Blood Flow Changes in Wilson’s Disease

**FIGURE 2:**
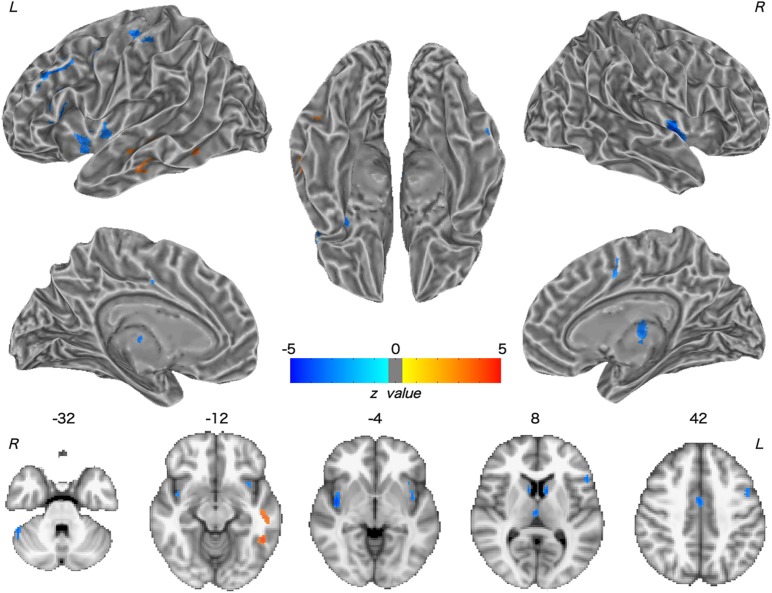
Group difference in CBF between WD and HC. The warm and cold colors denote significantly increased and decreased CBF in WD patients, respectively. CBF, cerebral blood flow. L, left; R, right.

**FIGURE 3:**
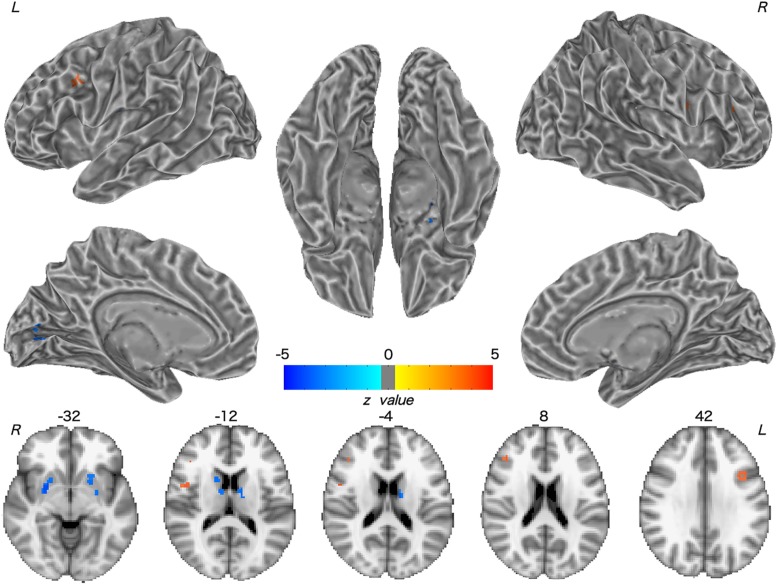
Group difference in FCS between WD and HC. The warm and cold colors denote significantly increased and decreased FCS in WD patients, respectively. FCS, functional connectivity strength. L, left; R, right.

Patients with WD showed decreased CBF in the bilateral insular and caudate, right cerebellum, left ventrolateral prefrontal cortex (VLPFC), precentral cortex, cingulate cortex, and thalamus, and increased CBF in the left middle temporal cortex (Figure 2 and Table 2). Compared with healthy controls, patients with WD exhibited decreased FCS in the bilateral lentiform and caudate nuclei, left cuneus, and right thalamus, and increased FCS in the bilateral dorsolateral prefrontal cortex (DLPFC; Figure 3 and Table 3).

**Table 2:** Group difference in CBF between WD and HC.

Region	Side	MNI coordinate			Voxel	*z* value
		*x*	*y*	*z*		
VLPFC (45)	L	-50	22	18	330	-4.88
MiTG	L	-54	-24	-12	83	3.94
PrG	L	-40	-18	54	109	-4.13
MCC	R	2	-2	42	89	-4.41
CRB	R	50	-46	-32	55	-4.12
Insular	L	-30	12	-16	95	-4.29
	R	42	-4	-4	145	-5.45
Caudate	L	-8	8	6	51	-4.30
	R	10	8	12	50	-4.23
Thalamus	R	2	-16	8	52	-4.14


*CBF, cerebral blood flow; VLPFC, ventrolateral prefrontal cortex; MiTG, middle temporal gyrus; PrG, precentral gyrus; MCC, middle cingulate cortex; CRB, cerebellum; L, left; R, right*.

**Table 3:** Group difference in FCS between WD and HC.

Region	Side	MNI coordinate			Voxel	*z* value
		*x*	*y*	*z*		
Lentiform	L	-22	10	-10	90	-3.99
	R	28	0	-6	122	-4.82
Caudate	L	-12	-6	18	32	-4.03
	R	12	20	6	50	-4.05
Thalamus	R	10	-4	14	21	-3.51
Ceneus	L	-14	-82	6	-3.96	-3.96
DLPFC (9)	L	-36	12	30	33	3.78
DLPFC (46)	R	42	32	20	21	3.81


*FCS, functional connectivity strength; DLPFC, dorsolateral prefrontal cortex*.

### Cerebral Blood Flow – Functional Connectivity Strength Coupling Changes in Wilson’s Disease

**FIGURE 4:**
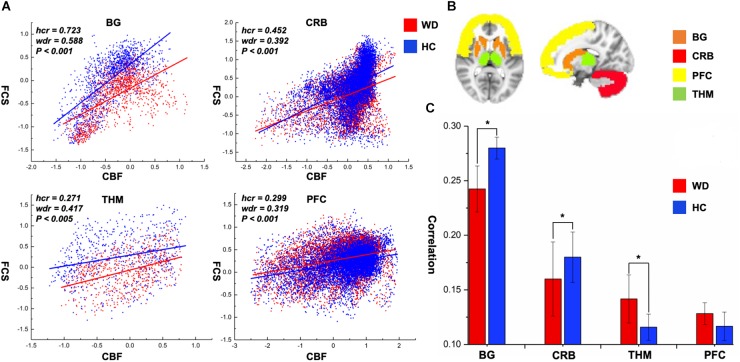
CBF–FCS coupling changes of WD in basal ganglia, cerebellum, thalamus, and prefrontal cortex. **(A)** Scatter plots of mean GM level spatial correlations across voxels between CBF and FCS in HC (blue) and WD (red), respectively. **(B)** The GM regions of BG, CRB, THM, and PFC were extracted from AAL template. **(C)** The bar graph means CBF–FCS correlation across subjects in HC and WD. ^∗^Significant differences between both groups (*P* < 0.05). BG, basal ganglia; CRB, cerebellum; THM, thalamus; PFC, prefrontal cortex; hcr, the relationship between FCS and CBF in healthy controls; wdr, the relationship between FCS and CBF in patients with Wilson’s disease.

CBF was significantly correlated with FCS within the whole GM, but the correlations in patients with WD were slightly lower than those in healthy controls (Figure 1). Patients with WD had significantly reduced CBF–FCS coupling in the basal ganglia and cerebellum, and increased CBF–FCS coupling in the thalamus relative to HC (Figure 4). There were no significant differences in CBF–FCS coupling in the prefrontal cortex.

## Discussion

To our knowledge, this is the first study to explore changed CBF–FCS coupling in WD by combining BOLD and ASL techniques. The whole GM CBF–FCS coupling in WD was slightly decreased compared to that of healthy controls. The WD group showed reduced CBF–FCS coupling in the basal ganglia and cerebellum, and increased coupling in the thalamus. Patients with WD exhibited decreased CBF and FCS in numerous brain regions, primarily the basal ganglia, and thalamus. These findings improve our understanding of the neural mechanisms underlying WD from the perspective of neurovascular coupling.

### CBF Changes in WD

Decreased CBF was observed in the bilateral caudate nucleus, right thalamus, and cerebellum in WD. One possible explanation for this observation is neurovascular decoupling in these regions. In the neurovascular coupling hypothesis (Kuschinsky, 1991), CBF changes are governed by neuronal activity and may reflect the altered neuronal activity in WD. For instance, brain regions with enhanced neuronal activity tend to have a greater brain metabolism, resulting in increased perfusion (Poornima et al., 2016). Because of the neuronal loss and atrophy in basal ganglia and thalamus, due to copper deposition in WD, the neuronal activity in these regions was reduced (Hu et al., 2017), which resulted in decreased CBF. These results are consistent with previous studies, which have identified decreased metabolic rate of glucose consumption and hypoperfusion in the putamen, caudate nucleus, thalamus, cerebellum, and frontal cortex (Piga et al., 2008; Huster, 2010; Ishida et al., 2012). Decreased CBF was also found in the left VLPFC and precentral cortex, right cingulate cortex, and bilateral insular cortex. The VLPFC is involved in executive functions and cognitive processes (Yang and Raine, 2009). The precentral cortex and insular are related to motor control (Fink et al., 1997; Meier et al., 2008) and multimodal sensory processing (Phan et al., 2002), whereas the cingulate cortex is involved in emotion formation and processing (Hadland et al., 2003). The basal ganglia and thalamus are highly interconnected with the cerebral cortex; for example, the prefrontal cortex is highly connected with the thalamus and parts of the basal ganglia (Silkis, 2001), and the cingulate cortex and insular directly receive inputs from the thalamus (Mcfarland and Haber, 2000; Ikemoto et al., 2015). All of these interconnections are mediated by the CBGTC (Silkis, 2001). CBF decreases in these regions may be the result of neurotransmitter (e.g., dopamine and GABA) changes in WD, because these chemicals also play a part in modulating vascular response (Cauli et al., 2004; Tayebati et al., 2011). The alterations in neurotransmitters may contribute to the disrupted CBGTC in WD, which results in imprecise regulation of CBF. In addition, patients with WD also exhibited a CBF increase in the left middle temporal cortex. One possible reason is that drug therapy may have effects on CBF in these regions. The increased CBF may be explained by the medication-induced dopamine turnover increase, resulting in CBF increase (Ala et al., 2007; Huster, 2010).

### FCS Changes in WD

FCS decreased in patients with WD relative to healthy controls, including in the bilateral lentiform and caudate nuclei, left cuneus, and right thalamus. Structural damage, such as GM reduction and white matter alterations, may engender dysfunctional connectivity within the human brain (Hu et al., 2017; Wang et al., 2017), resulting in aberrant information transmission. The use of both traditional MRI and diffusion tensor imaging techniques has demonstrated brain gray and white matter structural abnormalities in the basal ganglia, thalamus, and cerebral cortex (Page et al., 2004; Piga et al., 2008; Jadav et al., 2013). FCS describes the whole-brain functional connectivity profile of each voxel from a global network perspective and reflects the role of each voxel in information transmission within the whole-brain network (Liang et al., 2013). The precise coordination of inter-regional functional synchronization may be impaired because of the loss of structural integrity (Honey et al., 2007; Mikail et al., 2009), thus giving rise to FCS decrease in WD. Moreover, patients with WD also showed increased FCS in the bilateral DLPFC. The DLPFC is associated with executive functions, such as working memory, cognitive flexibility, planning, and inhibition (Yang and Raine, 2009). The cognition problems (e.g., impulsivity, promiscuity, executive dysfunction, slow cognition, and memory loss) and psychiatric problems (e.g., depression, anxiety, and psychosis) in WD might result from the joint abnormal functions of the frontal cortex and subcortical regions, such as the basal ganglia (Hu et al., 2017). Although the regions with increased FCS in this study might not be the basis of the cognitive and psychiatric problems of WD, the functional connectivity increase in WD might be explained as a compensatory mechanism; the human brain tends to generate a compensatory response to structural impairments (Appel-Cresswell et al., 2010; Palmer, 2010; Palmer et al., 2010).

### Neurovascular Abnormality in WD

The whole-brain correlation between CBF and FCS was computed in this study to identify the neurovascular coupling in WD. Neurovascular coupling depends on the integrity of neurovascular units, such as neurons, glial cells, and vascular components (Hawkins and Davis, 2005). The whole-brain CBF–FCS correlations in WD are slightly reduced relative to healthy controls, which may represent neurovascular decoupling in WD. Particularly, CBF–FCS correlations in the basal ganglia and cerebellum are significantly lower in WD than in healthy controls, possibly indicating neurovascular imbalance in WD. In the brain, astrocytes act as a bridge that links neural activity to the vascular response (Stobart and Anderson, 2013; Howarth, 2014). Abnormal astrocytes in the basal ganglia may reduce the connection between neural activity and vascular response, resulting in decreased CBF–FCS correlations. Interestingly, patients with WD exhibited a stronger CBF–FCS correlation in the thalamus than did healthy subjects. The increased CBF–FCS correlation may demonstrate that patients with WD improve synchronism between CBF and FCS in some brain regions, which is affected by the compensatory mechanism. However, this is the first time that CBF and FCS are used to study the neurovascular coupling in WD, and there is no literature on neurovascular coupling improving local brain regions. Therefore, the hypothesis here should be investigated in further studies.

## Conclusion

In conclusion, aberrant coupling between resting-state CBF and functional connectivity in WD was revealed in this study. Specifically, we found that decreased FCS and CBF predominately occurred in subcortical regions involved in sensory processing and motor regulation, including the basal ganglia and thalamus, and increased CBF and FCS occurred in regions involved in cognitive control and emotional modulation, including the temporal cortex and DLPFC. These findings highlight the neurovascular imbalance in WD, which may be a potential neural mechanism underlying the pathophysiology of WD.

## Limitations

In this study, we adopted CBF and FCS to investigate neurovascular coupling in patients with WD. Although the present study is meaningful, there are some limitations. Firstly, it is very important that the results are stable over time in a repeated series of experiments. Therefore, another relatively larger data set should be collected for validating the present study. Secondly, because of the relatively small paramagnetism of copper, copper deposition might influence the MRI signal on the basal ganglia nucleus (Kanda et al., 2016). A patient group should be added in future studies, which would receive copper drainage treatment with the copper content close to normal levels, as a control group to minimize the influence of copper deposition on MRI signals. Thirdly, many studies associated neuroimaging results with clinical parameters, which can serve as neuroimaging indicators for some neurological disease. In the current study, we did not collect cognitive behavior and clinical parameters. Future studies must explore the relationship between functional network deficits and clinical implication. Finally, we used a standard gray mask to carry out the neurovascular coupling analysis, without taking the problem of incomplete coverage of individual image acquisition into consideration. In future studies, a common group-level mask on resting fMRI and the ASL should be used for the CBF–FCS coupling analysis.

## Ethics Statement

This study was approved by the Human Research Committee in the First Affiliated Hospital of Anhui University of Chinese Medicine.

## Author Contributions

All authors listed have made a substantial, direct and intellectual contribution to the work, and approved it for publication.

## Conflict of Interest Statement

The authors declare that the research was conducted in the absence of any commercial or financial relationships that could be construed as a potential conflict of interest.

## Footnotes

1 http://www.fil.ion.ucl.ac.uk/spm

2 http://rfmri.org/DPARSF
